# The combined use of DTI and MR elastography for monitoring microstructural changes in the developing brain of a neurodevelopmental disorder model: Poly (I:C)-induced maternal immune-activated rats

**DOI:** 10.1371/journal.pone.0280498

**Published:** 2023-01-13

**Authors:** Lucy Liu, Andre Bongers, Lynne E. Bilston, Lauriane Jugé

**Affiliations:** 1 Faculty of Medicine & Health, University of New South Wales, Sydney, New South Wales, Australia; 2 Neuroscience Research Australia, Sydney, New South Wales, Australia; 3 Biological Resources Imaging Laboratory, University of New South Wales, Sydney, New South Wales, Australia; UCSI: UCSI University, MALAYSIA

## Abstract

Early neuropathology mechanisms in neurodevelopmental disorders are partially understood because routine anatomical magnetic resonance imaging (MRI) cannot detect subtle brain microstructural changes *in vivo* during postnatal development. Therefore, we investigated the potential value of magnetic resonance elastography (MRE) and diffusion tensor imaging (DTI) in a rat model of neurodevelopmental disorder induced by maternal immune activation. We studied 12 offspring of mothers injected with polyriboinosinic-polyribocytidylic acid (poly (I:C), 4 mg/kg) on gestational day 15, plus 8 controls. T2-weighted anatomical MR images, MRE (800 Hz) and DTI (30 gradient directions, b = 765.8 s/mm^2^, 5 images, b = 0 s/mm^2^) were collected when the rats were 4 and 10 weeks old, and results were compared with histological analysis performed at week 10. Ventricles were ~1.4 fold larger from week 4 in poly (I:C) rats than in controls. No other morphological abnormalities were detected in poly(I:C) rats. At week 4, larger ventricles were correlated with lower external capsule fractional anisotropy and internal capsule radial diffusion (Pearson, r = -0.53, 95% confidence intervals (CI) [-0.79 to -0.12], and r = -0.45, 95% CI [-0.74 to -0.01], respectively). The mean and radial diffusion of the corpus callosum, the mean and axial diffusion of the internal capsule and the radial diffusion properties in the external capsule increased with age for poly (I:C) rats only (Sidak’s comparison, P<0.05). Cortical stiffness did not increase with age in poly (I:C) rats, in contrast with controls (Sidak’s comparison, P = 0.005). These temporal variations probably reflected abnormal myelin content, decreased cell density and microglia activation observed at week 10 after histological assessment. To conclude, MRE and DTI allow monitoring of abnormal brain microstructural changes in poly (I:C) rats from week 4 after birth. This suggests that both imaging techniques have the potential to be used as complementary imaging tools to routine anatomical imaging to assist with the early diagnosis of neurodevelopmental disorders and provide new insights into neuropathology.

## Introduction

Neurodevelopmental disorders, such as autism or schizophrenia, are leading causes of morbidity in children [1]. Although psychiatric disorders typically emerge in late adolescence or young adulthood [2], slowly progressive and brain microstructural alterations have been reported before the clinical diagnosis of impairments [3].

Structural MRI has characterized enlarged lateral ventricles and smaller regional brain volumes in neurodevelopmental disorders [4–8]. However, structural brain MRI abnormalities have not been reported in all patients with neurodevelopmental disorders [9]. A hypothesized reason is that macroscopic parameters cannot capture early microstructural alterations that are too subtle to alter brain volumes. Developing an imaging protocol that can detect and monitor early brain microstructural changes *in vivo* during brain development may assist with early diagnosis. It may also be useful for developing early interventions and providing new insights into the neuropathology underlying mental health disorders in adults.

Diffusion tensor imaging (DTI) [10, 11] may provide valuable insight into neurodevelopmental disorders because white matter integrity has been related to cognitive development and long-term outcomes in adults [12, 13]. It has been recently used to identify compromised white matter structures in schizophrenia [14], depressive disorder [15], and autism spectrum disorder [16], with lower fractional anisotropy and higher diffusivity in patients compared to typically developing children. However, reports of longitudinal changes in diffusion properties of the white matter during the prodromal phase of the disease are lacking.

Magnetic resonance elastography (MRE) may be a useful technique to monitor gray matter microstructural changes during abnormal neurodevelopment and complement white matter assessment with DTI [17]. Brain stiffness, as measured by MRE, has been reported as a valuable imaging marker of abnormal gray matter structure during hydrocephalus development in juvenile and adult rats and can detect temporal changes during disease progression [18, 19]. Softer brain tissue has also been reported in adults and mice with neurodegenerative disease [20–22] and in children with cerebral palsy [23], suggesting it has the potential as a marker of loss of gray matter tissue integrity in neurodevelopmental disorders.

Therefore, this study aimed to investigate the potential value of the combined use of MRE and DTI in the early detection of the respective longitudinal microstructural changes in gray and white matter during postnatal development in a pre-clinical setting. We used a common rat model of neurodevelopmental problems after maternal immune activation [24] in combination with healthy age and gender-matched rats as a control group. Changes in MR diffusion properties (fractional anisotropy (FA), mean diffusion (MD), axial diffusion (AD) and radial diffusion (RD)) and MR elastography properties (complex shear modulus) were compared with histopathology at the end-point. Based on previous studies, we hypothesized that 1- increase in FA and decrease in MD, AD and RD with age would be more moderate in maternal infected rats than in controls, and 2- brain complex shar modulus, as measured by MRE, would decrease with age more in the rat model of maternal immune activation than in controls.

## Materials and methods

### Animals

This study was approved by the local animal care ethics committee (University of New South Wales, Australia, #18/52A). We studied 12 male offspring of female rats in whom immune activation was stimulated during pregnancy with an intravenous injection into the lateral tail vein of the viral mimic polyriboinosinic-polyribocytidylic acid (poly (I:C), 4 mg/kg, cat # P9582, lot # 118M4035V, Sigma-Aldrich, Castle Hill, Australia) to induce maternal cytokine responses, and alter neurodevelopment [24]. The injection was performed on gestational day 15 under anaesthesia using 1.5% isoflurane (cat # FGISO0250, Pharmachem, Eagle Farm, Australia) in 100% oxygen delivered at a rate of 1L/min via a nose cone. Rats exposed prenatally to the Poly (I:C) viral mimic develop progressive neurodevelopmental brain structure abnormalities [25]. As these abnormalities accumulate, behavioural alterations mimicking some of those seen in schizophrenia, autism, and depression typically emerge in rat’s adulthood [26]. Eight male control rats from saline-injected mothers following the same protocol were also included.

Four pregnant female Sprague-Dawley (ArcCrl:CD(SD)IGS) rats from Animal Resources Centre (Australia) were required for the study and acclimatized for one week before the commencement of the protocol. Pregnant rats gave birth in the local animal facility, and male pups were randomly selected from the four litters. Only male pups were selected because disease progression differs between genders, and males have greater ventricular enlargement over time in this animal model [25]. All rats were housed in cages on a 12-hour light/dark cycle with pellet food and water available ad libitum. Pups were housed with their mothers until three weeks old.

The study was powered to detect a difference in the change in the brain viscoelastic properties between the two groups over time (primary outcome), consistent with an 8% increase in rat brain complex shear modulus observed in compression-driven dementia of 4 week old rats, with a mean standard deviation of 15% [18]. By using analysis of variance tests with a power of 0.8, a significance level of 0.05 and a correlation among repeated measures of 0.85, a minimum sample size of 6 rats per group was required. The sample size was increased to 12 and 8 for poly (I:C) and control groups, respectively, to be conservative and allow for dropout if required.

### MR protocol

Rat pups were scanned 4 and 10 weeks after birth to characterize early and late neurodevelopmental processes, corresponding to a child and an adolescent human stages, respectively [27]. These time-points were chosen to occur before the emergence of abnormal behaviours mimicking schizophrenia in this rat model (~3 months old) [28], but when neuropathological and motor changes are becoming apparent. Cerebral enlargement has been observed in these rats as early as six weeks after birth, and motor alterations typically emerge when they are young adults (~10 weeks old) [25].

All rats were imaged at the Biological Resources Imaging Laboratory (Mark Wainwright Analytical Centre) of the University of New South Wales (Australia) using a horizontal 9.4T BioSpec Avance III 94/20 MR microimaging system (Bruker, Ettlingen, Germany). A 86 mm ID quadrature volume transmitter was used with a 20 mm diameter single loop surface receive coil (Bruker) placed on top of the rat head. Rats were positioned prone, headfirst in the scanner and anaesthetized with 0.5%-1.5% isoflurane (cat # FGISO0250, Pharmachem, Eagle Farm, Australia) in 100% medical oxygen at a constant rate of 1L/min via a face mask. Body temperature and respiratory rate were continuously monitored using an animal monitoring system (SA Instruments, Inc., Stony Brook, NY, USA). A heated blanket was used to maintain body temperature between 36°C—37°C.

**Fig 1:**
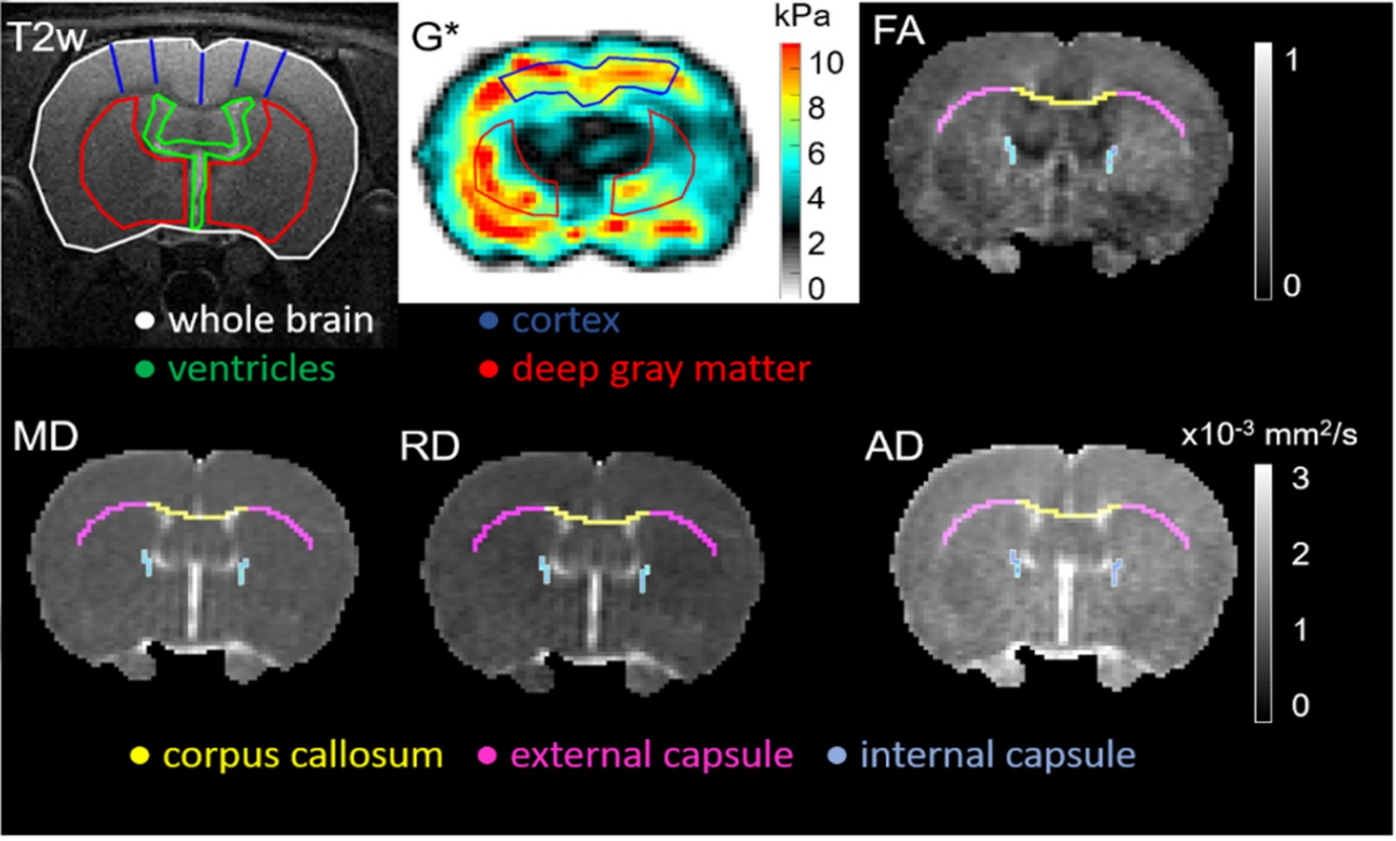
Typical images/maps obtained with this MRI protocol. Anatomical T2 weighted (T2W) image; shear modulus (G*) map, as measured by MRE at 800 Hz; fractional anisotropy (FA), mean diffusivity (MD), axial and radial diffusivity (AD and RD) maps, as measured with DTI (Bregma -0.72 mm (29)). Anatomical images were used to measure the cross-sectional areas of the ventricular system (green), whole-brain (white), and deep gray matter (red), along with the cortical thickness in the middle slice acquired (navy blue). The cortical thickness was averaged over 5 locations on the “roof” of the lateral ventricles. The magnitude of the complex shear modulus (G*) was calculated for the cortex (navy blue) and deep gray matter (red). FA, MD, RD and AD were obtained for the corpus callosum (yellow), external (pink) and internal (purple) capsules.

All images were collected in axial orientation centred on the foramina of Monro (Bregma -0.72 mm [29], Fig 1). The precise location was identified at the start of the imaging protocol with a rapid anatomical scan (separate from the images used for the data analysis). The parameters used were: TR/TE = 3300/10.7 ms, Effective TE = 42.7 ms, RARE factor = 8, 4 averages, 31 contiguous slices of 1 mm thickness, FOV = 25.6 × 25.6 mm, in-plane resolution = 200 × 200 μm, acquisition time = 3 min 3s.

T2-weighted anatomical MR images were collected using a 2D fast spin-echo sequence (TurboRARE) with the following parameters: TR/TE = 3300/8.5 ms, Effective TE = 42.7 ms, RARE factor = 8, 8 averages, 9 contiguous slices of 300 μm thickness, FOV = 19.2 × 19.2 mm, in-plane resolution = 150 × 150 μm, acquisition time = 8 min 21s. These images were used to assess the development of macroscopic anatomical abnormalities in poly (I:C) rats over time.

A DTI protocol was used to quantify white matter (corpus callosum, internal and external capsules) diffusion properties [18]. The Diffusion protocol used a Steijskal-Thanner diffusion preparation scheme with a four-shot echo-planar image readout with the following parameters: TR/TE = 2500/23.5 ms, 4 averages, 1 repetition, 30 gradient directions with a b factor = 765.8 s/mm^2^ and 5 b0 reference images, 3 continuous slices of 900 μm thickness, in-plane resolution = 200 × 200 μm, FOV = 25.6 × 25.6 mm, acquisition time = 23 min 20 s. Saturation slices were not used in this DTI protocol to prevent aliasing artefacts because the FOV was more than double the radius of the planar surface receive coil used, and the signal intensity rapidly drops beyond one radius from the centre of the coil (i.e. 10 mm) [30].

MR elastography was used to measure cortical and deep gray matter shear modulus (G*) as previously [18, 19, 31]. Sinusoidal vibrations at 800Hz were generated by an electromagnetic shaker (Brüel & Kjaer, Nærum, Denmark) located outside the scanner and synchronized with the imaging. A carbon fibre rod was connected at one end to the shaker to the rat’s incisors at the other end, allowing vibration to propagate through the skull and into the brain tissue. MR elastography data was obtained using a modified two-dimensional multi-slice spin-echo sequence with sinusoidal motion sensitized gradients (570 mT/m) synchronized with the continuous sinusoidal mechanical vibration of 800 Hz. Brain tissue displacements were three-dimensionally phase-encoded over eight time-points distributed equally over one vibration period: TR/TE = 1880/28.75 ms, 1 average, 24 repetitions, FOV = 19.2 × 19.2 cm, 9 continuous slices of 300 μm thickness, in-plane resolution = 300 × 300 μm, acquisition time 57 min 54 s.

Approximately 2 hours per rat were required for collecting the entire imaging protocol, including set-up time.

### MR analysis

T2-weighted anatomical images were used to measure the cross-sectional areas of the ventricular system, whole brain and deep gray matter, along with the cortical thickness in the middle slice acquired. The cortical thickness was measured from the pial surface to the subcortical gray matter and averaged over 5 locations on the “roof” of the lateral ventricles. Regions of interest were manually outlined using ImageJ (version 1.52, National Institutes of Health, USA) (Fig 1) by a single researcher, following the same protocol as previously reported [18].

MR diffusion metrics (MD, FA, RD and AD) of the white matter were calculated on a pixel-wise basis using DSI Studio (http://dsi-studio.labsolver.org, February 2019 version, Department of Biomedical Engineering, Carnegie Mellon University, PA, USA) by calculating eigenvectors of the diffusion matrix after correction for the motion and Eddy current artefacts. Mean MR diffusion measures were obtained for the corpus callosum, external and internal capsules of each rat brain also over the middle slice (Fig 1). For each rat and at each time point, regions of interest were drawn manually on the b0 images and exported to the MD map, where voxels with a MD > 1.8 × 10^−3^ mm^2^/s were excluded from the analysis to avoid potential cerebrospinal fluid partial volume effect. FA, MD, RD and RD were extracted based on these regions of interest. The background around the brain tissue was masked out to increase the reconstruction efficacy and facilitate further visualization.

The magnitude of the complex shear modulus (G*), a measure of brain stiffness, was calculated by numerically solving the wave equation for a linear viscoelastic medium [32] using a validated custom software [33, 34]. MRE data were considered reliable if the local ‘non-linearity, a measure of noise in the displacement data [35], was less than 18% in the cortex and less than 25% in the deep gray matter [36]. G* of the cortex and deep gray matter were averaged over the middle three of the nine acquired slices (Fig 1). Regions of interest were drawn manually on the magnitude images and then exported on the stiffness map. Pixels adjacent to the ventricles were excluded to avoid the partial volume effect of the cerebrospinal fluid.

### Histology and immunohistochemistry

Following the final scan at week 10, all rats were euthanized via intracardiac perfusion of phosphate-buffered saline (cat # P4417, Sigma-Aldrich, Castle Hill, Australia) followed by 10% buffered formalin (cat HT501128, Sigma-Aldrich, Castle Hill, Australia) under deep anaesthesia with 3% Isoflurane (cat # FGISO0250, Pharmachem, Eagle Farm, Australia). Rat brains were extracted and immersed in formalin for at least 48h at 4°C before being stored in 70% ethanol also at 4°C (Sigma-Aldrich, Castle Hill, Australia).

For the histological analysis, the brains were embedded in paraffin and cut in 4 μm thick axial sections centred on the foramina of Monro using a Leica RM2135 Rotary Microtome (Leica Biosystems, Wetzlar, Germany). A large portion of each brain was sectioned during the sample preparation. Only the slices at the exact level of the Foramina of Monro were stained to ensure the best location matching possible between MR data and the histology. Two slices were obtained from each brain. Tissues were prepared, stained and imaged at the Biomedical Imaging Facility of the University of New South Wales (Australia).

**Fig 2:**
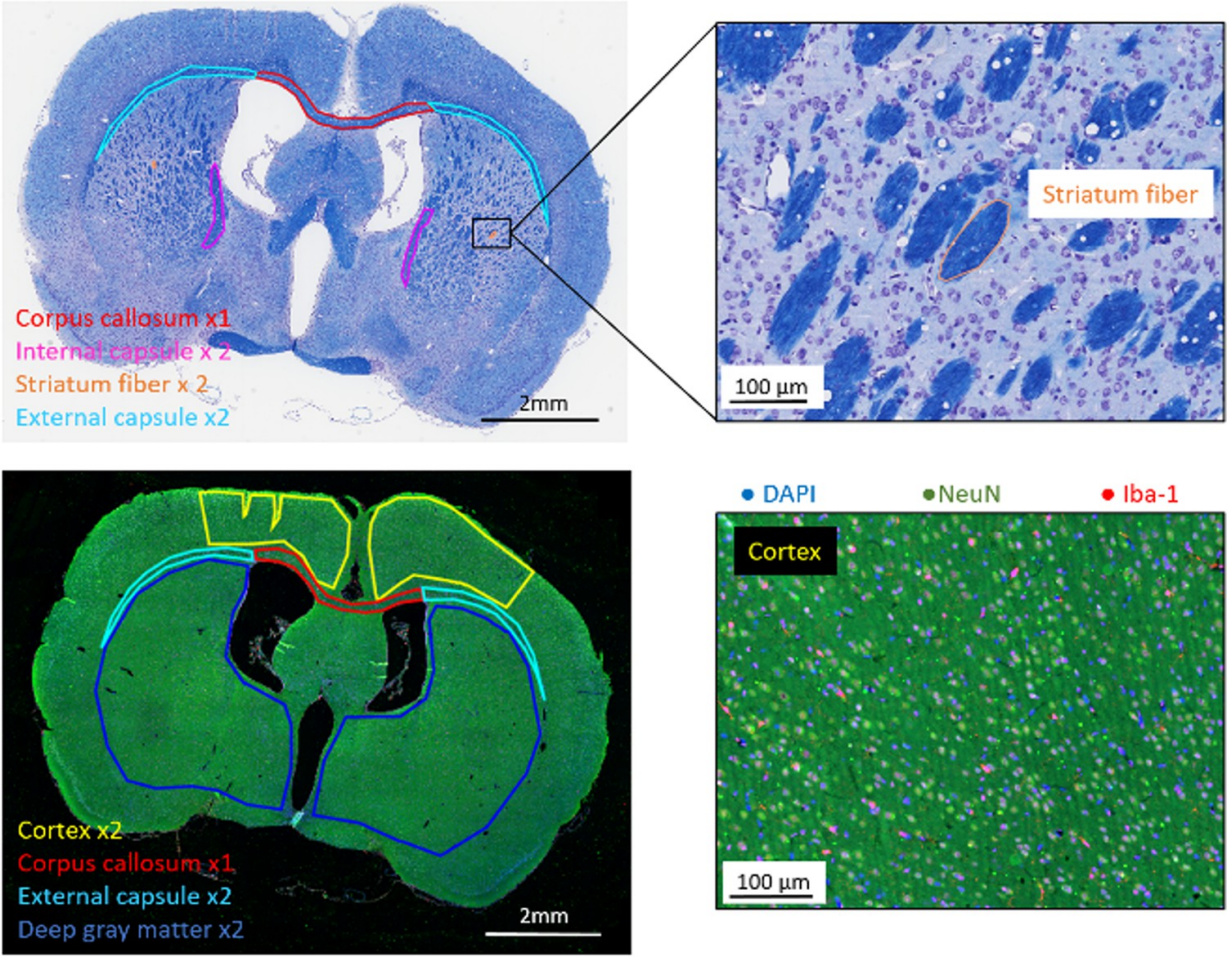
Typical histological sections of a poly (I:C) rat brain stained with luxol fast blue and cresyl violet (top row) and with Iba-1 (red), NeuN (green) and DAPI (blue) (bottom row) at 10 weeks after birth. Regions of interest are shown on the histological sections.

The first slice was stained with Luxol Fast Blue (cat # 2460610, BDH Chemicals, Kilsyth, Australia) and Cresyl Violet (cat # C-5042, lot # 129H3732, Sigma-Aldrich, Castle Hill, Australia), following standard protocols. It was used to determine white matter myelin content [37] and neuronal density [38] at week 10, and to investigate the effect of prenatal immune activation on the difference in myelin content of the striatofugal fibers crossing the globus pallidus nucleus, a major component of the basal ganglia within the deep gray matter (Fig 2).

The second slice was stained with goat anti-rat Iba-1 polyclonal antibody (1:200, cat # ab5076, lot # GR3190885-3) with the secondary donkey polyclonal anti-goat IgG—H&L red—fluorescent antibody (1:200, cat # ab150131, lot # GR3245238-1) and mouse anti-rat NeuN monoclonal antibody (1:10, cat # ab104224, lot # GR3255973-4) with the secondary goat polyclonal anti-mouse IgG- H&L green-fluorescent antibody (1:200, cat # ab150105, lot # GR3212997-1) to quantify the microglial/macrophages (hereafter termed “microglia” for simplicity) and neuronal content, respectively (all from Abcam, Cambridge, MA, USA) (Fig 2). Before staining, slices were dewaxed with two baths of 5 min in Xylene (cat # UN1307, Ajax Finechem, ThermoFisher, Waltham, MA, USA), three baths of 1 min in 100% ethanol (cat # UN1170, Ajax Finechem, ThermoFisher, Waltham, MA, USA), one bath of 1 min in 70% (v/v) ethanol, and finally rinse in Milli-Q water for 1 min. Antigen retrieval was done with pH 6.0 antigen retrieval buffer (citrate, cat # K8005, Agilent Dako, Santa Clara, CA, USA) for 5 min at 110°C. Blocking was done in 0.1 M Glycine in Tris Buffered Saline (cat # K8007, TBS, Agilent Dako, Santa Clara, CA, USA) for 30 min at room temperature. Primary antibodies were incubated for 1 hour at room temperature. After three washes of 2 min with TBS, the secondary antibodies were incubated for 30 minutes at room temperature, and slices were finally rinsed twice in TBS for 2 min each time. Iba-1 and NeuN fluorescent slides were also co-stained with DAPI (4′,6-diamidino-2-p0 henylindole, cat # FP1490A, PerkinElmer, Waltham MA, USA) blue-fluorescent, to stain nuclei and aid visualization on imaging (2 drops in 1ml for 5 min). Slices were then rinsed with TBS for 2 min and purified water before being coverslipped with Prolong mounting medium (cat # P36930, Invitrogen, Carlsbad, CA, USA) and then stored 4°C.

One additional control and one other poly (I:C) male rat brain were obtained from the litter of pups and stained following the same protocols to determine the myelination and cell density at four weeks after birth. These two rats did not undergo the MRI scan at this time point.

### Histology and immunohistochemistry analysis

Luxol fast blue/cresyl violet stained slides were scanned using an Aperio ScanScope XT Scanner with standard line scanning at 40× magnification, resulting in a spatial resolution of 0.25 μm/pixel (Leica Biosystems, Wetzlar, Germany). Digital slide scans were subsequently visualized and analyzed using QUPATH (version 0.2.0, Queen’s University, Belfast, Ireland) [39]. Captured images were first analyzed using a positive staining detection algorithm developed in the software for myelin quantification (downsample factor 1.0, Gaussian sigma 0.2 μm, Alcyan blue positive threshold 0.25 OD/units), and then with a cell detection algorithm (analyzed pixel size 0.5 μm, background Radius 8.0 μm, median filter 1.0 μm, sigma filter 1.5 μm, min nuclear area: 10.0 μm^2^, max nuclear area: 400.0 μm^2^, Hematoxylin Eosin threshold: 0.15 OD/units). Regions of interest were manually drawn with a polygonal tool over the corpus callosum and each left and right external and internal capsules, excluding tissue tears and folding (Fig 2). Additionally, one fibre bundle randomly selected from each right and left striatum of the basal ganglia was also segmented. The final histological measurements for the external and internal capsules and the striatum fibre were obtained by averaging results from the two regions of interest drawn in each structure. Myelin content was expressed as the percentage of the total area of each region of interest that stained positive for myelin. Cell density represents the number of cell bodies staining positive with cresyl violet per millimetre square.

NeuN and Iba-1 stained brain slices were imaged on Vectra Polaris (Akoya Biosciences) slide scanner at 40× magnification objective with the following filters: DAPI–Exc 391/32 Em 453/30, Alexa 488 –Exc 486/30 Em 532/18, Alexa 647 –Exc 649/50 Em 704/30 (PerkinElmer, Waltham, USA). The scanned slide images were imported into QUPATH slide analysis software. Watershed cell detection algorithms were optimized on a minimum of 5 images for each staining to segment cells and obtain cell counts of DAPI, NeuN and Iba-1 stained cells. The detection pixel size was set to 0.5μm to increase the detection accuracy. An 8μm background radius was determined using the software ImageJ to subtract the background. To improve the detection of labelled cells, image texture reduction was applied in the form of a median filter (MF), and the noise was reduced using a sigma filter (SF) as follows: DAPI MF 0μm SF 0.8μm, NeuN MF 1.5μm SF 1.5μm, Iba-1 MF 1μm SF 1.5μm. The minimum area of the detected object was determined (DAPI 10μm^2^ NeuN 30μm^2^ and Iba-1 15μm^2^), and the maximum was kept at 400μm^2^ for all labels. The object detection threshold was determined by analyzing 10 images (DAPI 5 OD/units, NeuN 7 OD/units, and Iba-1 17 OD/units). Two regions of interest were manually drawn with a polygonal tool over each of the left and right deep gray matter, two regions over both the left and right cortex, one region over the corpus callosum and two more regions over the external capsules, excluding tissue tears and folding (Fig 2). The internal capsule could not be segmented on these slides as it could not always be reliably identified, in contrast with luxol fast blue stained slices where there is a clear distinction between grey and white matter. The final histological measurements for the deep gray matter, cortex, and external capsule were obtained by averaging results from the two regions of interest drawn in each structure. Cell density represents the number of cell bodies staining positive with DAPI per millimetre square. Microglial density was calculated from the ratio of Iba-1 positive cells to the total cells (i.e. those stained with DAPI) and the neuronal density from the ratio of NeuN positive cells to the total cells. Both are presented as percentages.

### Statistical analysis

Statistical tests were performed with GraphPad Prism (version 8.3.0, San Diego, California, USA) or SPSS (version 28.0.1.0I, BM SPSS Statistics, Chicago, Illinois, USA). P values of less than 0.05 were considered significant. Group results at 4 and 10 weeks are reported as mean ± standard deviation. They were analyzed with a repeated measure two-way ANOVA or by fitting a mixed model [40] if there were missing values, both followed by a Sidak’s multiple comparisons test. Histological measurements at week 10 were analyzed with an independent sample t-test (2-tailed) with Welch’s correction. Correlations between ventricle size and anatomical measurements, DTI and MRE metrics at weeks 4 and 10, and histological results (only for week 10) were assessed using Pearson correlations since most data passed the Shapiro-Wilk normality test. For each time-point, the Pearson correlations were calculated across both the controls and poly (I:C) rats. Estimates and their 95% confidence intervals are reported in the manuscript. Bonferroni adjusted confidence intervals to account for the multiple comparisons are reported in the S1 Table.

## Results

All animals were healthy for the duration of the experiment and gained weight with age. There was no difference in weight between controls and poly (I:C) rats at week 4 (114 ± 28g for both groups), but at week 10, controls were heavier than poly (I:C) rats (480 ± 54g and 425 ± 64g, respectively, P_time×group_ = 0.04).

### Anatomical abnormalities

**Fig 3:**
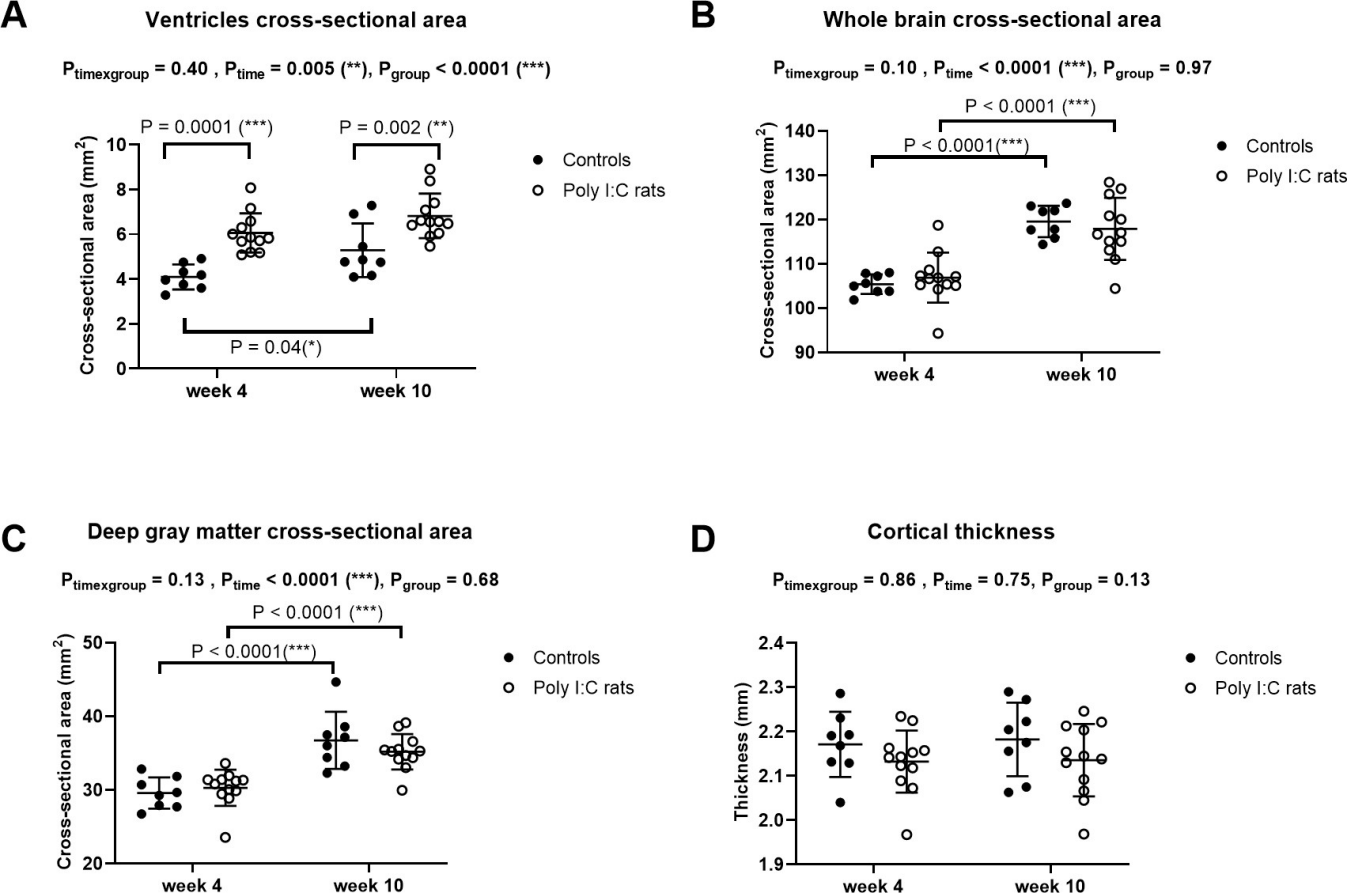
Anatomical abnormalities. Cross-sectional areas of the ventricles (A), whole-brain (B), deep gray matter (C), and cortical thickness (D) measured in controls (n = 8, ●) and poly (I:C) rats (n = 12, ○) at week 4 and week 10 after birth (mean ± standard deviation). Data were analyzed with a repeated two-way ANOVA with Sidak’s multiple comparisons.

The cross-sectional area of the ventricles was significantly larger in poly (I:C) rats than in controls at weeks 4 and 10 (Fig 3A, P_group_ < 0.0001), even though ventricles enlarged significantly with age in the controls, but not in poly (I:C) (Sidak’s multiple comparisons, P = 0.04, and P = 0.12, respectively). Ventricles were 1.48 fold larger in average from week 4 in poly (I:C) rats than in controls.

The cross-sectional area of the whole brain and deep gray matter increased with age in both groups but did not differ between cohorts (Fig 3B and 3C, P_time_ < 0.0001 for both). Cortical thickness was not different between groups and did not change with age (Fig 3D).

### White matter–MR diffusion properties

**Fig 4:**
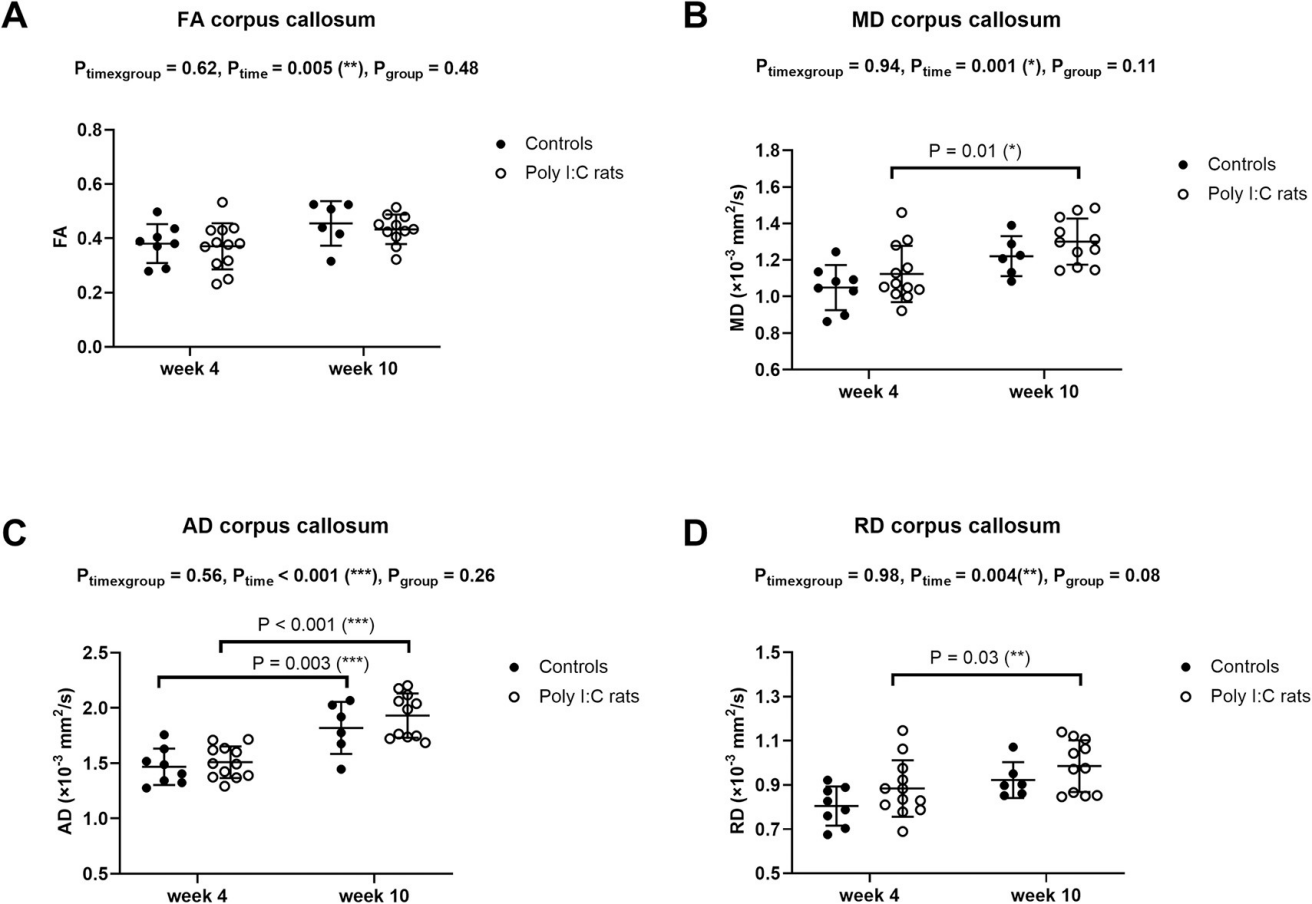
Corpus callosum MR diffusion properties (mean ± standard deviation) in controls (●) and poly (I:C) rats (○) measured at 4 and 10 weeks after birth. (A) Fractional anisotropy—FA, (B) Mean diffusivity–MD, (C) Axial diffusivity–AD, and (D) Radial diffusivity–RD. MD, RD and AD increased with age in poly (I:C) rats, while only AD increased from week 4 to week 10 in controls. FA of the corpus callosum did not differ between groups and did not change with age. Data were analyzed with a mixed-effects model with Sidak’s multiple comparisons.

**Fig 5:**
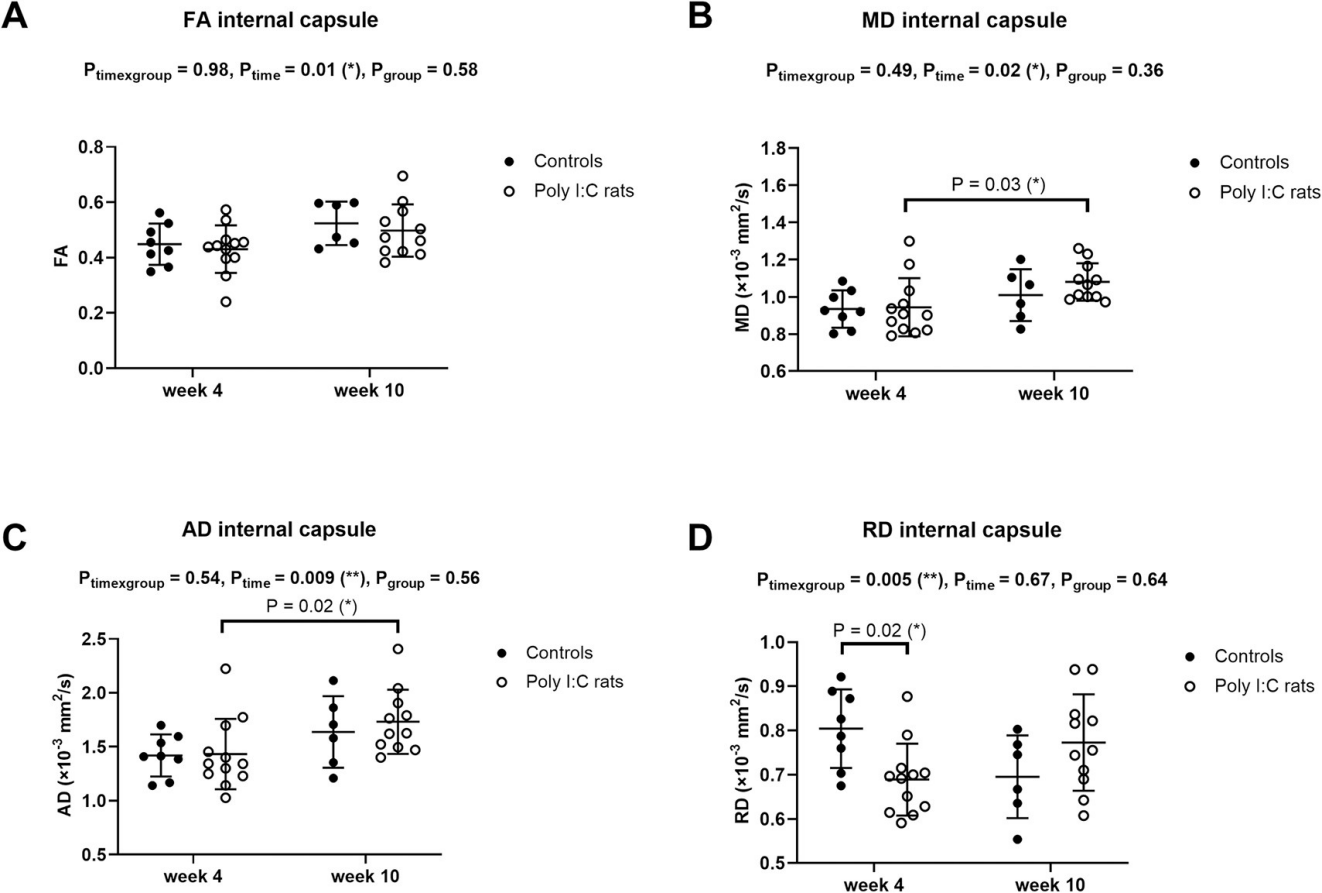
Internal capsule MR diffusion properties (mean ± standard deviation) in controls (●) and poly (I:C) rats (○) measured at 4 and 10 weeks after birth. (A) Fractional anisotropy—FA, (B) Mean diffusivity–MD, (C) Axial diffusivity–AD, and (D) Radial diffusivity—RD. MD and AD increased with age only in poly (I:C) rats. RD was lower in poly (I:C) rats at week 4. Data were analyzed with a mixed-effects model with Sidak’s multiple comparisons.

**Fig 6:**
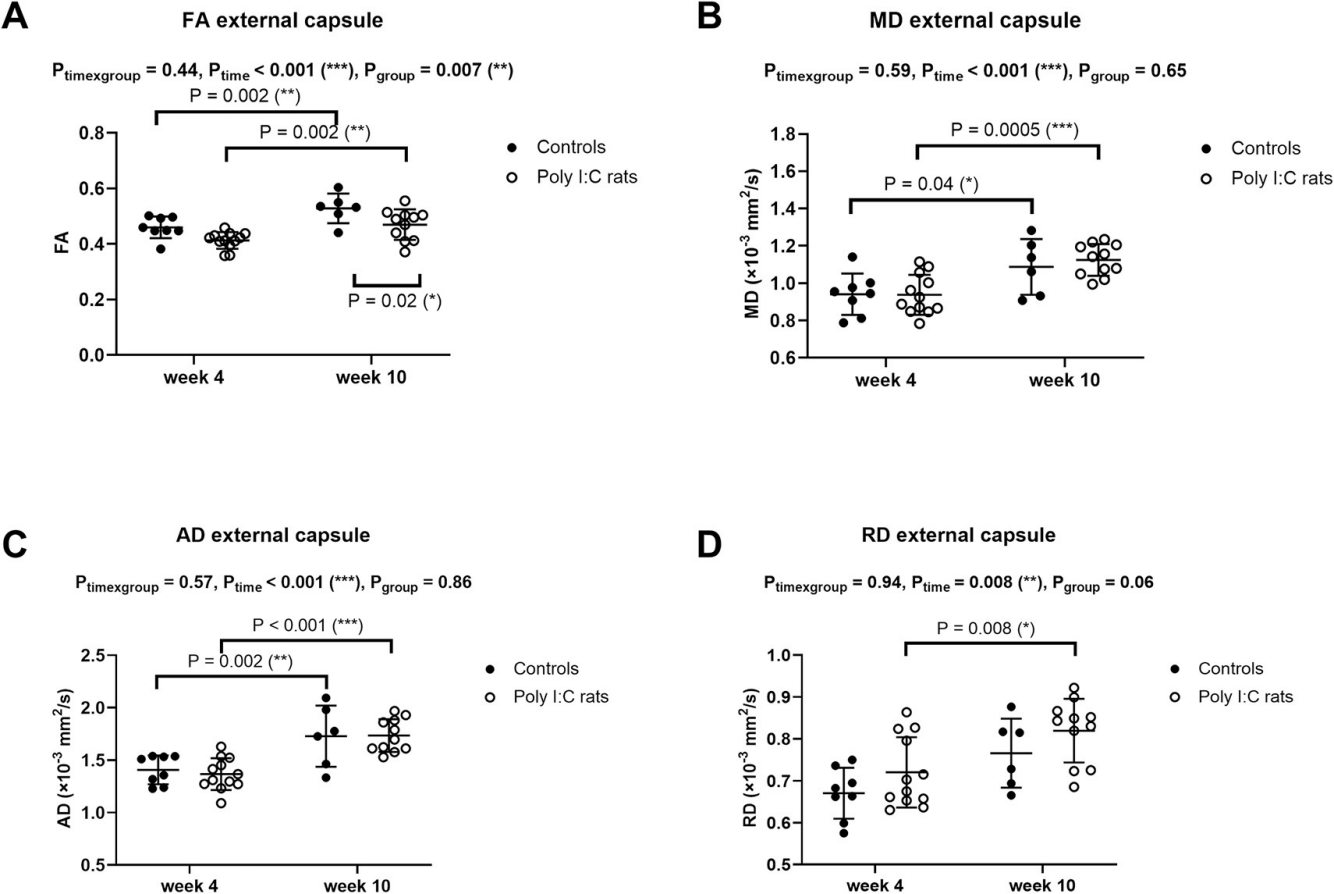
External capsule MR diffusion properties (mean ± standard deviation) in controls (●) and poly (I:C) rats (○) measured at 4 and 10 weeks after birth. (A) Fractional anisotropy—FA, (B): Mean diffusivity–MD, (C) Axial diffusivity–AD, and (D) Radial diffusivity–RD. Increases in all four MR diffusion parameters with age was only seen in poly (I:C) rats. Data were analyzed with a mixed-effects model with Sidak’s multiple comparisons.

MR diffusion measurements from two controls (of 8) and one poly (I:C) rat (of 12) at week 10 were excluded due to large motion artefacts related to head movement that severely degraded image quality. All MR diffusion measurements at week 4 were included in the further analysis. MD, AD, and RD of the corpus callosum (Fig 4), MD and AD of the internal capsule (Fig 5), and FA, MD, RD, AD of the external capsule (Fig 6) increased with age. This was more commonly seen in poly (I:C) rats, but measurements did not differ significantly between groups at either time point, except for FA of the external capsule at week 10 (Fig 6A, P_group_ = 0.007). RD of the internal capsule was significantly higher in controls than poly (I:C) rats at week 4 (Fig 5D, Sidak’s multiple comparisons, P = 0.02), but not at week 10, and did not significantly change with age. Only for RD of the internal capsule, there was a significant interaction between age and group (Fig 5D, P_time×group_ = 0.005). Other MR diffusion variables measured in the white matter of controls and poly (I:C) rats did not differ between groups or change with age.

### Gray matter–MR elastography properties

**Fig 7:**
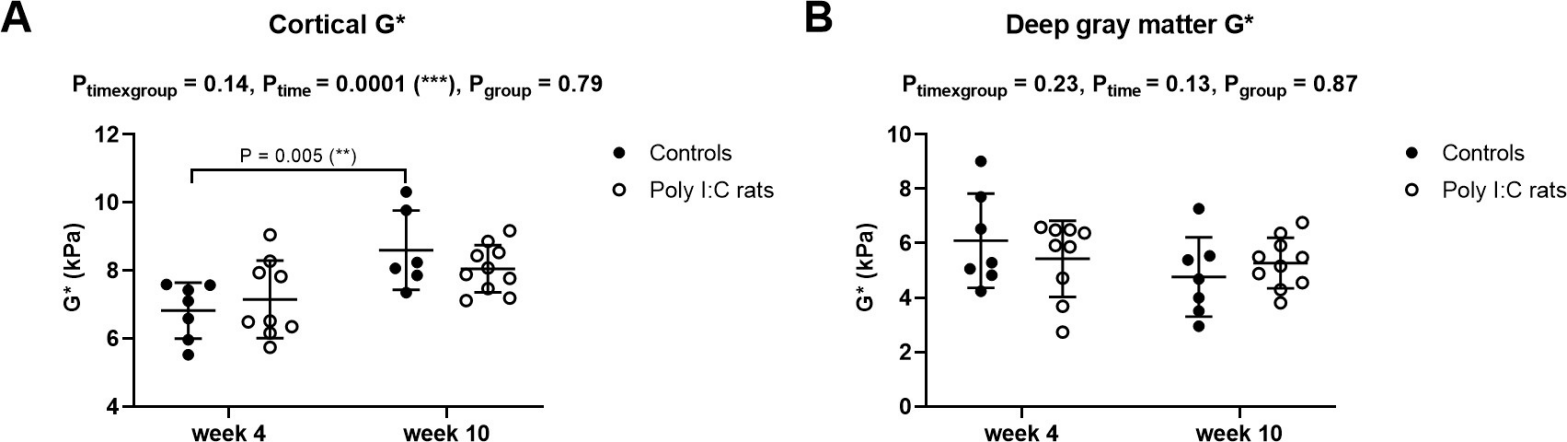
Shear modulus (G*)(mean ± standard deviation) of the cortex (A) and deep gray matter (B), measured at 800Hz in controls (●) and poly (I:C) rats (○) at weeks 4 and 10 after birth. Cortical G* increased from week 4 to week 10 only in control rats. Data were analyzed with a mixed-effects model with Sidak’s multiple comparisons. P values for significant Sidak’s comparisons are reported.

Fifteen of 80 total brain stiffness (G*) measurements were excluded due to poor data quality (high ‘non-linearity’). Cortical stiffness in controls was significantly higher at week 10 than week 4, but not in poly (I:C) rats (Fig 7A). Deep gray matter stiffness did not differ between groups and between time points (Fig 7B). There was no significant interaction between age and groups for stiffness in either gray matter region.

### Histology

Myelin and microglial density in the corpus callosum and external capsule were higher in poly (I:C) rats than in controls at week 10. At the same time, the cell density (i.e. determined from cresyl violet staining) was lower in poly (I:C) rats than in controls (Table 1). There was no microstructural difference between groups in the internal capsule.

**Table 1:** Histological measurements.

	Week 4		Week 10		Welch’s t-test at week 10
	Control (n = 1)	Poly (I:C) rat (n = 1)	Controls (n = 8)	Poly (I:C) rats (n = 12)	P values
**Corpus callosum**					
Myelin density (%)	-	-	84 ± 5	91 ± 1	P = 0.03 (*)
			(n = 5)	(n = 6)	
Cell density (CV+ cells/mm^2^)	3383	2953	2518 ± 203	2188 ± 215	P = 0.003 (**)
Microglial density (%)	3.5	4.3	8.5 ± 2.6	22.7 ± 16.1	P = 0.02 (*)
			(n = 7)	(n = 11)	
**Internal capsule**					
Myelin Density (%)	-	-	80 ± 7	88 ± 8	P = 0.14
			(n = 4)	(n = 6)	
Cell density (CV+ cells/mm^2^)	2934	2728	2633 ± 434	2649 ± 680	P = 0.95
			(n = 6)	(n = 11)	
**External capsule**					
Myelin (%)	-	-	86 ± 2	92 ± 1	P < 0.001 (***)
			(n = 5)	(n = 6)	
Cell density (CV+ cells/mm^2^)	3398	3305	2287 ± 224	2034 ± 180	P = 0.02 (*)
Microglial Density (%)	10.0	15.9	8.9 ± 2.4	25.1 ± 15.6	P = 0.007 (**)
**Cortex**				(n = 11)	
Cell density (DAPI+ cells / mm^2^)	3840	3635	3696 ± 638	3515 ± 330	P = 0.24
				(n = 11)	
Neuronal density (%)	11.1	15.4	45 ± 7	41 ± 10	P = 0.40
				(n = 11)	
Microglia density (%)	3.3	3.9	3.9 ± 0.6	10.0 ± 7.7	P = 0.03 (*)
				(n = 11)	
**Deep gray matter**					
Cell density (DAPI + cells/ mm^2^)	4018	3600	3196 ± 197	3403 ± 395	P = 0.48
			(n = 5)	(n = 8)	
Neuronal density (%)	14.4	24.8	31 ± 14	31 ± 13	P = 0.97
			(n = 5)	(n = 8)	
Microglial density (%)	4.1	6.3	5.4 ± 0.4	9.6 ± 2.3	P = 0.11
			(n = 5)	(n = 8)	
Striatum fibre myelin (%)	-	-	93 ± 5	98 ± 1	P = 0.12
			(n = 5)	(n = 6)	

Mean ± standard deviation of the percentage of myelin and nuclei content observed in the white matter in 8 controls and 12 poly (I:C) rats 10 weeks after birth (except if n reported differently). * indicates a significant difference between groups at week 10 (Welch’s test). Histological measurements were obtained from one additional control rat and one other poly (I:C) rats at week 4. Results from 29 tissue samples and a further 45 luxol fast blue stained samples were unavailable due to technical problems with tissue slicing and staining. Microglial density is not available for the internal capsule, because it could not be segmented on NeuN and Iba-1 stained brain slices as it could not always be reliably identified. Abbreviation: cresyl violet (CV).

The microglial percentage was higher in the cortex of poly (I:C) rats than in controls at week 10, but not in the deep gray matter (Table 1). Cell density and neuronal percentage did not differ between groups in both gray matter regions at week 10.

### Estimates of correlations with ventricle size at weeks 4 and 10

**Fig 8:**
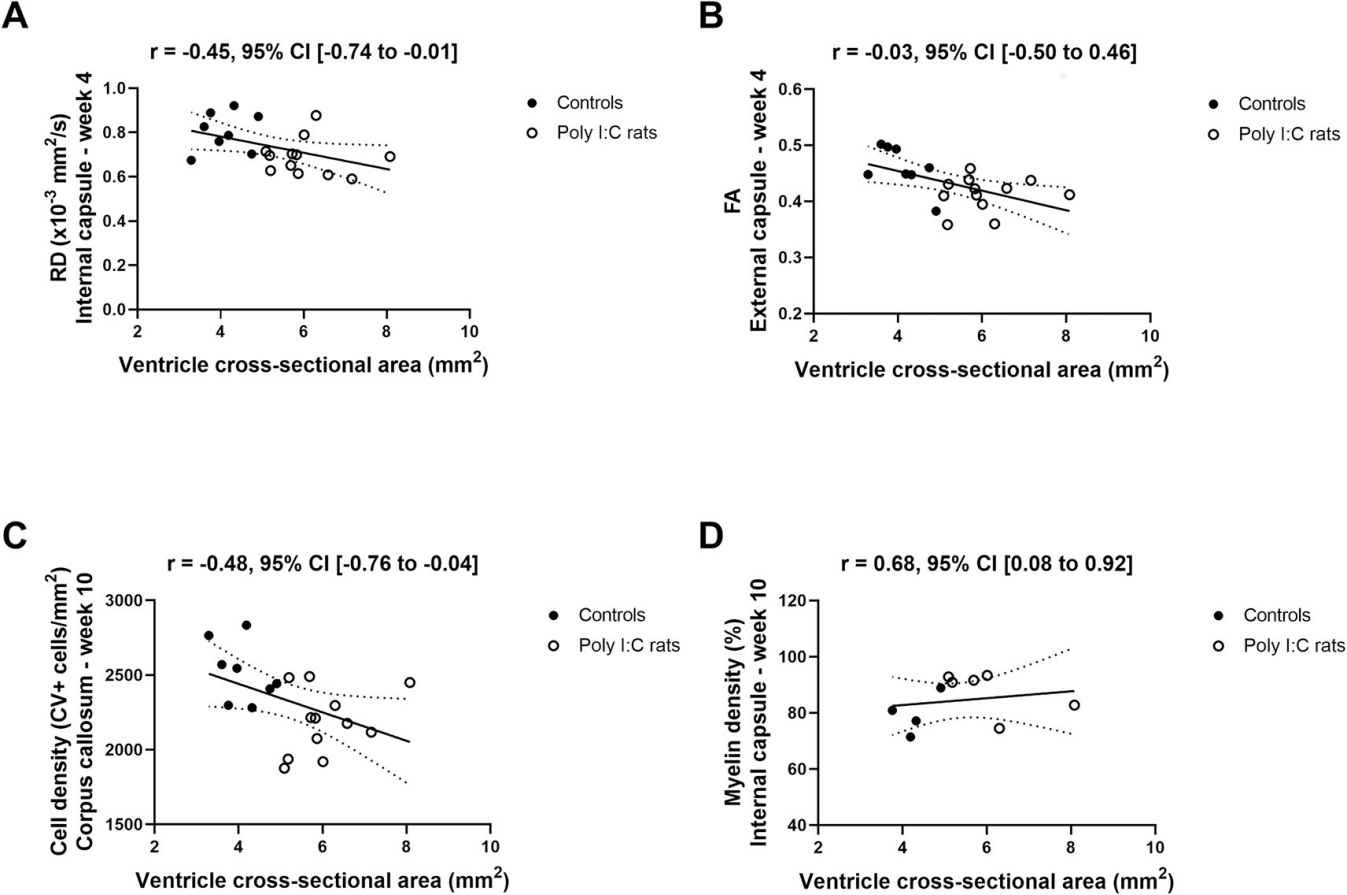
Estimates of the Pearson correlations with ventricle cross-sectional area and their 95% confidence intervals (CI). Radial diffusivity (RD) in the internal capsule at week 4 (A) and fractional anisotropy (FA) in the external capsule at week 4 (B); cell density at week 10 in the corpus callosum (C) and myelin density at week 10 in the internal capsule (D) across the whole sample. Control (●) and poly (I:C) rats (○) are indicated. Bonferroni-adjusted confidence intervals are reported in the S1 Table.

Ventricle size was not correlated with the cortical thickness or cross-sectional areas of the whole brain and deep gray matter at either time point. However, at week 4, larger ventricles were correlated with lower RD of the internal capsule and FA of the external capsule (Fig 8A and 8B, respectively), but not in the corpus callosum. Others DTI measurements at week 4 were not correlated with ventricle enlargement. At week 10, no significant correlations between ventricle size and DTI measurements were observed in all three white matter regions. Ventricle cross-sectional area was also not correlated with gray matter stiffness at weeks 4 and 10. Finally, at week 10, a larger ventricle cross-sectional area was correlated to a smaller cell density in the corpus callosum and higher myelin percentage in the internal capsule (Fig 8C and 8D, respectively). All estimates and unadjusted 95% confidence intervals of the Pearson correlations are reported in Table 2. Bonferroni-adjusted confidence intervals are reported in the S1 Table.

**Table 2:** Estimates of the correlations with ventricle size.

Pearson correlations with ventricle size (r, unadjusted 95% confidence interval)	Week 4	Week 10
**Anatomical variables**		
Cortical thickness	-0.23 [-0.61 to 0.23]	-0.16 [-0.56 to 0.30]
Whole brain cross-sectional area	0.26 [-0.20 to 0.63]	0.24 [-0.22 to 0.62]
Deep gray matter cross-sectional area	0.08 [-0.38 to 0.50]	0.12 [-0.34 to 0.43]
**DTI**		
**FA**—Corpus callosum,	-0.37 [-0.70 to 0.08],	0.05 [-0.45 to 0.52],
Internal capsule,	-0.10 [-0.52 to 0.35],	0.19 [-0.32 to 0.62],
External capsule	-0.53 [-0.79 to -0.12]	-0.03 [-0.50 to 0.46]
**MD**—Corpus callosum,	0.25 [-0.22 to 0.62],	0.33 [-0.18 to 0.70],
Internal capsule,	0.03 [-0.42 to 0.47],	0.27 [-0.24 to 0.66],
External capsule	0.08 [-0.38 to 0.50]	0.26 [-0.29 to 0.54]
**RD**—Corpus callosum,	0.40 [-0.05 to 0.72],	0.26 [-0.25 to 0.66],
Internal capsule,	-0.45 [-0.74 to -0.01],	0.03 [-0.46 to 0.50],
External capsule	0.30 [-0.16 to 0.66]	0.23 [-0.29 to 0.64]
**AD**—Corpus callosum,	-0.01 [-0.45 to 0.43],	0.30 [-0.21 to 0.68],
Internal capsule,	-0.02 [-0.46 to 0.43],	0.30 [-0.21 to 0.68],
External capsule	-0.11 [-0.52 to 0.35]	0.24 [-0.28 to 0.54]
**MRE**		
G*—Cortex	0.39 [-0.13 to 0.74]	-0.18 [-0.62 to 0.35]
G*—Deep gray matter	-0.08 [-0.55 to 0.43]	-0.19 [-0.61 to 0.32]
**Histology**		
**Corpus callosum**—Myelin density,	-	0.57 [-0.05 to 0.87]
Cell density,		-0.48 [-0.76 to -0.04]
Microglial density		0.21 [-0.28 to 0.62]
**Internal capsule**—Myelin density,	-	0.68 [0.08 to 0.92]
Cell density		-0.35 [-0.70 to 0.14]
**External capsule**—Myelin density,	-	0.44 [-0.22 to 0.82]
Cell density,		-0.34 [-0.68 to 0.12]
Microglial density		0.38 [-0.13 to 0.73]
**Cortex**—Cell density,	-	-0.05 [-0.49 to 0.42]
Neuronal density,		-0.22 [-0.61 to 0.26]
Microglia density		0.08 [-0.39 to 0.51]
**Deep gray matter**—Cell density,	-	0.36 [-.24 to 0.76]
Neuronal density,		0.08 [-0.49 to 0.60]
Microglia density,		0.23 [-0.36 to 0.69]
Striatum fibre myelin		0.43 [-0.23 to 0.82]

Estimates with unadjusted 95% confidence intervals of the Pearson correlations were calculated across control and treated rats between ventricle cross-sectional area and anatomical variables, DTI and MRE measurements at weeks 4 and 10, and histological results at week 10. Abbreviations: Diffusion Tensor Imaging (DTI); Fractional Anisotropy (FA); Mean Diffusivity (MD); Axial and Radial Diffusivity (AD and RD); Magnetic Resonance Elastography (MRE); Shear modulus (G*). Bonferroni-adjusted confidence intervals are reported in the S1 Table.

## Discussion

### Main results

This study demonstrates that adding MRE and DTI to routine anatomical imaging has potential to improve the early detection of microstructural changes in white and gray matter tissue during postnatal development in a rat model of maternal immune activation. This is demonstrated by the three key results from this study. Firstly, although ventricular enlargement was visible from week 4 in poly (I:C) rats, ventricular expansion did not progress with age, and no morphological abnormalities were detected in those rats over time, indicating that anatomical measures alone do not provide insight into disease progression. Secondly, most diffusion measurements (FA, MD, AD and RD) increased across the white matter in the poly (I:C) rats, while only FA of the external capsule, AD of the corpus callosum and external capsule and MD of the external capsule increased with age in controls, indicating that DTI may aid the assessment of subtle white matter microstructural changes induced by prenatal maternal immune activation during brain maturation from 4 weeks after birth. Third, cortical stiffness did not increase with age in poly (I:C) rats in contrast with controls, indicating that MRE was able to detect microstructural deterioration associated with this gray matter neurodevelopmental disorder and separate them from age-related changes.

### Anatomical variations

Brain changes in pups from poly (I:C) treated mothers were consistent with the previous characterization of this model with moderately larger ventricle cross-sectional area in those rats at both ages, compared to controls [25]. Cortical and striatal neurodevelopment have been reported to be abnormal in poly (I:C) rats during the prodromal phase, leading to behavioural abnormalities in young adult rodents [25]. However, these abnormalities, along with ventricular expansion, were not sufficient to produce observable brain tissue reduction. Indeed, the poly (I:C) rats in this study followed the typical brain maturation trajectory, where significant tissue volume gain and no change in cortical thickness were observed between 3 weeks and 2 months after birth [41].

### White matter

DTI detected white matter microstructural alterations in poly (I:C) rats as early as four weeks after birth. Lower fractional anisotropy of the external capsule and radial diffusivity of the internal capsule were measured in the presence of larger ventricles at week 4, possibly related to white matter demyelination and loss of tissue integrity. Four weeks after birth may also be the earliest time at which DTI can sense microstructural abnormalities in this animal model since fractional anisotropy and mean diffusivity were not different between treated rats and controls at 3 weeks after birth in another study [42]. However, further investigations are required to confirm this because that study was performed on *ex vivo* fixed brains, which are known to have altered diffusion properties [43].

Radial diffusion is thought to be the most sensitive DTI metric to myelination changes (41). As such, a lower radial diffusivity of the internal capsule in the poly (I:C) rats compared to controls at week 4 probably reflects myelin abnormalities in those rats. Poly (I:C) mice have been reported to suffer from delayed myelination in the hippocampus at 2 weeks after birth that was not detected later when the mice were 9 weeks old [44]. If this also occurs in the internal capsule over our time frame, this may explain why no difference in myelin between groups of rats was observed in our histological analysis at week 10. Further observation over time might be useful to delineate the time course of these changes, along with the use of alternative markers of myelin content (e.g. quantitative MRI techniques [45], myelin basic protein [46, 47]) to verify myelin measurements and understand the relationship between the radial diffusion of the internal capsule and the neuropathology of poly (I:C).

Most diffusion metrics in the white matter of controls did not change with age in this study, probably because by week 4 in healthy rats, neuronal proliferation and migration along with apoptosis and axonal pruning are completed [48]. Fractional anisotropy of the external capsule increased with age in controls, maybe driven by myelination processes consistent with observations that the corpus callosum fractional anisotropy increased after weeks 3 in rats [49]. Corpus callosum and external capsule axial diffusivity also increased with age in controls, as in the white matter of healthy humans with ageing [50], where it is thought to reflect axonal shrinkage [51].

In contrast with controls, significant increases in diffusion parameters were observed in all white matter regions studied for poly (I:C) rats. In particular, the mean and radial diffusion of the corpus callosum, the mean and axial diffusion of the internal capsule and the radial diffusion of the external capsule increased with age only for poly (I:C) rats. The neuropathological changes in Poly (I:C) rats didn’t have the anticipated effects on the DTI longitudinal changes. We thought that the lower density observed in pathological studies of the poly (I:C) model would likely reduce the age-related changes in DTI metrics during neurodevelopmental disorders. However, our results did not observe this. The effect of the lower cellular density in poly (I:C) rats on DTI metrics was likely offset by the higher myelin content in those rats observed at week 10 for the corpus callosum and the external capsule. However, this needs to be verified with appropriate histological analysis over this timeframe. Anisotropic diffusion properties have a limited ability to identify the processes causing change. They are determined by a combination of many factors, including myelination, inflammation, cell density and axon diameter [52]. Nevertheless, the results suggest that DTI can track widespread longitudinal microstructural alterations in poly (I:C) rats.

### Gray matter

Cortical stiffness did not increase with time in poly (I:C) rats, as seen in controls, which may reflect the progressive loss of tissue integrity after prenatal maternal immune activation and microglial activation. Microglia are softer than neurons [53], and the increased microglial density in the poly (I:C) rats compared to controls at week 10 could potentially explain brain tissue softening. Brain inflammatory response to maternal infection is thought to play a key role in the pathophysiology of this animal model that leads to schizophrenia-like symptoms [54]. Microglial activation was also reported in 40 and 60 days old (~6 and 9 weeks) poly (I:C) rats [55].

The increase in cortical stiffness with age in controls differs from studies in healthy humans that found either higher cortical stiffness in adolescents than in adults [56] or no age-dependency [35]. This may be due to the MRE vibration frequency used here. Human brain MRE is typically performed at a lower frequency (<80 Hz) than in rodents (here 800Hz) for technical reasons related to spatial resolution and vibration wavelength. Because the brain tissue is a viscoelastic material and stiffness increases with frequency, a difference in frequency-dependence of the brain tissue mechanical properties between children and adults may explain this difference in results. That is, if the frequency dependence is stronger in adults than in children over a frequency range of ~30–800 Hz. But this needs to be confirmed because no difference in frequency dependence between children, adolescents and adults was observed between 30 and 60 Hz [35]. Brain stiffness measured in this study is consistent with previous measurements of healthy rat brain tissue mechanical properties using the same technique at the same frequency 4 [18, 31] and 10 [19] weeks after birth.

The absence of longitudinal differences in stiffness between controls and poly (I:C) rats in the deep gray matter may reflect the lack of significant microstructural differences between groups at week 10. Total cell, neuronal, and microglia density and myelin content within the striatum fibre bundle did not differ between groups at week 10. However, it has been shown that striatum bundles increase in numbers between 3 weeks and 3 months after birth in rats [57], but this was not captured in this study and the effect of myelin on brain tissue mechanical properties is still debatable on whether it stiffen or soften the tissue [34, 58]. Furthermore, more variability in data collection into deeper brain regions than in the cortex due to an increased wave propagating attenuation [18] could also contribute to the absence of longitudinal changes.

#### The combined use of MRE and DTI

Individually, neither MRE nor DTI provides a complete picture of the tissue microstructural alterations occurring in neurodevelopmental disorders, though they are both sensitive to different microstructural changes with age. Therefore, our protocol combining MRE, DTI and routine anatomical imaging is a significant advancement over single-model studies, in line with current research efforts to promote multimodal neuroimaging protocols [17].

Common DTI methods, using a pure mono-exponential model, are often used to assess gray matter tissue microstructure changes, despite the minimal tissue anisotropy in the cortex and deep gray matter [59]. Therefore, these DTI-based imaging biomarkers have limited capacity to capture complex microstructural changes at the early stage of a disease in those regions of the brain, which may be better assessed using MRE. Also, anisotropic mechanical properties, as measured with the combined use of MRE and DTI [60, 61], are thought to be more sensitive to microstructural changes in anisotropic tissues [61].

### Limitations

This study had some limitations. First, the exclusion of 15 stiffness measurements due to high non-linearity is the result of the attenuation of the mechanical shear wave induced by the skull and the cerebrospinal fluid. This is a common problem in the brain MRE technique [18]. Second, since the study was primarily designed to investigate changes in mechanical properties and diffusion rather than changes in structural anatomy, cross-sectional areas of the corpus callosum, external and internal capsule were not investigated in this study, as this would have required a longer scan time to enable sufficient resolution. Third, DTI data from 3 rats at week 10 were excluded due to severe movement artefacts that could not be corrected. Data collection at the time of the scan could not be repeated, as ethical approvals restricted the duration of anaesthesia. Fourth, the mechanical properties of the white matter and hippocampus were not determined, even though there is some indication that they may change in these regions’ during growth in schizophrenia [17, 62]. This was not possible because the in-plane spatial resolution of the MRE scan was too low compared to the size of these structures. A higher spatial resolution would have substantially increased scan time and greatly increased risks for the animals under anaesthesia. Also, reconstruction of the brain shear modulus, G*, was based on the assumption that the tissue was isotropic in this study. While there are conflicting data on the presence and extent of mechanical anisotropy in the brain, this effect is known to be small. Five, this study was not designed to assess the effects of neuroplasticity or connectivity changes that might occur as compensation for neurodegeneration, but this would be fascinating to explore in future studies, perhaps using functional connectivity measures [63]. Sixth, volumetric brain measurements could also contribute to the discussion about the changes in cross-sectional brain areas observed over time. However, these measurements could not be validly done on the T2 weighted anatomical scans collected as only 9 slices of 300 μm thickness were acquired. It was also not possible to add a dedicated acquisition due to the limited time a rat can be kept under anaesthesia safely (~ 2 hours per rat). Finally, data collection was only performed at the foramina of Monro level (Bregma -0.72 mm), so regional differences outside the acquisition plane could not be assessed.

### Conclusion

This prospective study provides proof of concept and preliminary evidence that MRE and DTI can be used as complementary imaging tools to routine anatomical imaging to detect early abnormal white and gray matter microstructural changes that were too subtle to induce tissue shrinkage in a rat model of maternal immune activation. This multimodal approach has the potential to assist with early diagnosis, provide new insights into the neuropathology underlying mental health disorders in adults, and be useful for the development of future treatment. It also remains to be determined if these techniques are equally useful in humans.

## Supporting information

S1 Table Estimates of the correlations with ventricle size and their Bonferroni-adjusted confidence intervals. Estimates with Bonferroni-adjusted confidence intervals of the Pearson correlations were calculated across control and treated rats between ventricle cross-sectional area and anatomical variables, DTI and MRE measurements at weeks 4 and 10, and histological results at week 10. Abbreviations: Diffusion Tensor Imaging (DTI); Fractional Anisotropy (FA); Mean Diffusivity (MD); Axial and Radial Diffusivity (AD and RD); Magnetic Resonance Elastography (MRE); Shear modulus (G*).
(DOCX)
