# Supplementary material for: The combined use of DTI and MR elastography for monitoring microstructural changes in the developing brain of a neurodevelopmental disorder model: Poly (I:C)-induced maternal immune-activated rats
Source: PLoS One. 2023 Jan 13;18(1):e0280498. doi: 10.1371/journal.pone.0280498 (PMC9838869; doi:10.1371/journal.pone.0280498)
Supplement: S1 Table — Estimates with Bonferroni-adjusted confidence intervals of the Pearson correlations were calculated across control and treated rats between ventricle cross-sectional area and anatomical variables, DTI and MRE measurements at weeks 4 and 10, and histological results at week 10. Abbreviations: Diffusion Tensor Imaging (DTI); Fractional Anisotropy (FA); Mean Diffusivity (MD); Axial and Radial Diffusivity (AD and RD); Magnetic Resonance Elastography (MRE); Shear modulus (G*). (DOCX) [file pone.0280498.s001.docx]

**S1 Table: Estimates of the correlations with ventricle size and their Bonferroni-adjusted confidence intervals.**

| **Pearson correlations with ventricle size** (r, Bonferroni adjusted confidence interval) | **Week 4**  (99.7% confidence intervals) | **Week 10**  (99.8% confidence intervals) |
| --- | --- | --- |
| **Anatomical variables** |  |  |
| Cortical thickness | -0.23 [-0.70 to 0.37] | -0.16 [-0.66 to 0.43] |
| Whole brain cross-sectional area | 0.26 [-0.34 to 0.71] | 0.24 [-0.36 to 0.70] |
| Deep gray matter cross-sectional area | 0.08 [-0.85 to 0.60] | 0.12 [-0.47 to 0.63] |
| **DTI** |  |  |
| **FA** - Corpus callosum,  Internal capsule,  External capsule | -0.37 [-0.77 to 0.23],  -0.10 [-0.62 to 0.48],  -0.53 [-0.84 to 0.03] | 0.05 [-0.57 to 0.63],  0.19 [-0.46 to 0.71],  -0.03 [-0.61 to 0.58] |
| **MD** - Corpus callosum,  Internal capsule,  External capsule | 0.25 [-0.36 to 0.71],  0.03 [-0.53 to 0.57],  0.08 [ -0.50 to 0.60] | 0.33 [-0.34 to 0.77],  0.27 [-0.39 to 0.74],  0.26 [-0.40 to 0.74] |
| **RD** - Corpus callosum,  Internal capsule,  External capsule | 0.40 [ -0.19 to 0.78],  -0.45 [-0.80 to 0.14],  0.30 [-0.30 to 0.73] | 0.26 [-0.40 to 0.74],  0.03 [-0.58 to 0.62],  0.23 [-0.43 to 0.72] |
| **AD** - Corpus callosum,  Internal capsule,  External capsule | -0.01 [-0.56 to 0.55],  -0.02 [-0.56 to 0.54],  -0.11 [-0.62 to 0.48] | 0.30 [-0.36 to 0.76],  0.30 [-0.36 to 0.76],  0.24 [-0.42 to 0.73] |
| **MRE** |  |  |
| G* - Cortex | 0.39 [-029 to 0.81] | -0.18 [-0.71 to 0.49] |
| G* - Deep gray matter | -0.08 [-0.66 to 0.56] | -0.19 [-0.71 to 0.346] |
| **Histology** |  |  |
| **Corpus callosum** - Myelin density,  Cell density,  Microglial density | - | 0.57 [-0.26 to 0.91],  -0.48 [-0.82 to 0.10],  0.21 [-0.42 to 0.71] |
| **Internal capsule** - Myelin density,  Cell density | - | 0.68 [-0.15 to 0.95],  -0.35 [-0.77 to 0.29] |
| **External capsule** - Myelin density,  Cell density,  Microglial density | - | 0.44 [-0.41 to 0.88],  -0.34 [-0.75 to 0.27],  0.38 [-0.28 to 0.80] |
| **Cortex** - Cell density,  Neuronal density,  Microglia density | - | -0.05 [-0.60 to 0.53],  -0.22 [-0.70 to 0.40]  0.08 [-0.51 to 0.62] |
| **Deep gray matter** - Cell density,  Neuronal density,  Microglia density,  Striatum fibre myelin | - | 0.36 [-0.41 to 0.83]  0.08 [-0.63 to 0.71]  0.23 [-0.52 to 0.78]  0.43 [-0.42 to 0.89] |

Estimates with Bonferroni-adjusted confidence intervals of the Pearson correlations were calculated across control and treated rats between ventricle cross-sectional area and anatomical variables, DTI and MRE measurements at weeks 4 and 10, and histological results at week 10. Abbreviations: Diffusion Tensor Imaging (DTI); Fractional Anisotropy (FA); Mean Diffusivity (MD); Axial and Radial Diffusivity (AD and RD); Magnetic Resonance Elastography (MRE); Shear modulus (G*).
